# A comparison of breastfeeding among Han, Uygur and other ethnic groups in Xinjiang, PR China

**DOI:** 10.1186/1471-2458-6-196

**Published:** 2006-07-27

**Authors:** Fenglian Xu, Colin Binns, Guli Nazi, Lin Shi, Yun Zhao, Andy Lee

**Affiliations:** 1 Medical College, Shihezi University; Xinjiang, 832002, PR China; 2 School of Public Health, Curtin University of Technology, 6845, Western Australia; 3 Yumin County Hospital, Xinjiang, 834601, PR China; 4 Manas County Hospital, Xinjiang, 832000, PR China

## Abstract

**Background:**

Breastfeeding is an important factor in infant health and there have been no previous studies of breastfeeding practices in the different ethnic groups of this region of China. We aimed to compare breastfeeding rates and duration between Han, Uygur and other ethnic groups living in Xinjiang, PR China.

**Methods:**

A longitudinal study of infant feeding practices was undertaken using a sample that included different ethnic groups. Mothers were randomly recruited and interviewed in hospitals and after discharge were contacted in person or by telephone at approximately monthly intervals to obtain details of infant feeding practices. Setting: Xinjiang Uygur Autonomous Region, PR China. Subjects: A total of1219 mothers (578 Han, 360 Uygur and 281 'other minority' mothers) who delivered babies during 2003 and 2004 were interviewed in five hospitals or institutes located in both urban and rural areas.

**Results:**

'Any breastfeeding' rates in Han, Uygur and 'other minority' groups at discharge were 88.5 %, 94.3 % and 97.1 % respectively, and at six months 76.7 %, 54.7 % and 87.6 % respectively. While 'exclusive breastfeeding' rates in the Han, Uygur and 'other minority' groups at discharge were 78.0 %, 34.5 % and 83.1 % respectively, at six months they had fallen to 4.8 %, 0.4 % and 16.8 % respectively. The median duration of 'Exclusive breastfeeding' of Han, Uygur and 'other minority' babies were 1.5, 0.1 and 2.5 months respectively. The Uygur babies were least likely to be 'exclusive breastfed'.

**Conclusion:**

Uygur babies were least likely to be 'exclusive breastfed' and continued to 'any breastfeed' at six month. The 'any breastfeeding' rates in the Han group were lower in the first four months. An education program focused on breastfeeding continuation and exclusive breastfeeding is necessary in Xinjiang, especially for Uygur and Han ethnic groups.

## Background

Breastmilk provides the basis for the best nutrition for infants and brings health and development benefits to both babies and mothers [1,2]. 'Exclusive breastfeeding' for the first six months of life and continued breastfeeding up to two years of age or beyond are recommended by World Health Organization (WHO) [3].

Over the past forty years, China has experienced considerable changes in breastfeeding practices. After the emergence of modern China in the 1950's and 1960's, 'ever breastfed' rates in both urban and rural areas were over 80%. But during the 1970's, the rates started to decline considerably, especially in larger cities, because the availability and use of breastmilk substitutes became widespread and mothers went to work. For example, the 'ever breastfed' rate in the urban areas of China was 42.7% in 1975 and decreased to 33.6% in 1985 [4-6]. While the 'ever breastfeeding' rate in a rural area near Shanghai was 80% in early 1980's it had fallen to 44.1% by the early 1990's [7]. While the trends towards declining breastfeeding were similar in urban and rural areas, however the decline happened in urban areas a few years earlier and the rates in cities were relatively lower.

To address the decline in breastfeeding, the Chinese government set a national target of an 'exclusive breastfeeding' rate at four months of 80% by 2000 in the Chinese Children's Development Plan in 1990's [8]. As a result there were many efforts to promote breastfeeding and the breastfeeding rate in China started to increase in 1990's [9]. The breastfeeding initiation rate in Tianjin, one of the larger cites in China, was 55% in 1985, rising to 85% in 1991 and 95% in 1992 [9]. In a cohort study undertaken in 1997 in the city of Wuhan the 'any breastfeeding' rate was 95% at one month and 83% at four months; while the 'exclusive breastfeeding' rate was 88% at one month and 56% at four months respectively [10]. The mean duration of 'any breastfeeding', in a cross-sectional survey in 2002, was 6.3 months in Guangdong province, 7.7 months in Beijing, 8.2 months in Zhejiang province, 9.8 months in Hubei province and 12.3 months in Shandong province [11]. The longest breastfeeding mean duration report from China is in Tibet where it was 14.2 months in 1999 [12]. The general pattern in China is that the less developed areas had a relatively longer breastfeeding duration.

China is the most populous country in the world and with 56 ethnic groups. The largest group, the Han ethnic, make up over 92% of China's population. The remaining 55 ethnic groups are collectively called minorities,

The Xinjiang Uygur Autonomous Region (Xinjiang AR) in North-western China borders eight countries: Russia, Kazakhstan, Kirghizastan, Tajikistan, Pakistan, Mongolia, India and Afghanistan. There are more than 13 ethnic groups living in this area. Among them, the Uygur people account for 46%, Han 40% and Kazakh 7%. The population of Xinjiang had reached 19.6 million by the end of 2004. The average birth rate was 16 per thousand and death rate 5.1 per thousand.

The cultural factors that influence breastfeeding practices are acknowledged in the literature and have been widely reviewed [13-15]. A cross-sectional study undertaken in the Han ethnic population in Shihezi, Xinjiang in 1994–1996 found a breastfeeding rate of 34% at four month [16]. This was well below Chinese and international targets. However no studies have been undertaken on the breastfeeding rates in the minority groups in the province. The comparison between ethnic groups is needed to provide evidence to identify whether specific breastfeeding interventions are needed. The objective of this study was to compare breastfeeding rates and duration between Han ethnic Uygur and 'other minority' groups in Xinjiang, PR China through the first six months after birth.

## Methods

A longitudinal cohort study of breastfeeding practices was undertaken in the Xinjiang Uygur Autonomous Region, PR China. Mothers who delivered babies during 2003 and 2004 were interviewed while in hospital and were invited to participate in the study. The mothers were assured that all of the personal data collected would be kept confidential. After their return home mothers were contacted in person or by telephone at approximately monthly intervals (at 0.5, 1.5, 2.5, 3.5, 4.5 and 6 months respectively) and asked to complete a structured questionnaire to obtain details of breastfeeding practices.

A total of 1256 mothers were recruited from five hospitals and institutes located in urban areas(Shihezi People's Hospital, Shihezi Maternal and Child Health Care Institute, Urumqi Maternal and Child Health Care Institute) and rural areas (Chabuchaer Maternal and Child Health Care Institute and Yumin County Hospital) of the province. These hospitals and institutes covered majority of mothers who delivered babies in the cities and suburbs. About 70% mothers in Shihezi city and suburb delivered babies in Shihezi People's Hospital (about 50%) and Shihezi Maternal and Child Health Care Institute(about 20%). More than half Uygur babies in Urumqi were born in Urumqi Maternal and Child Health Care Institute. More than 50% babies in Chabuchaer and more than 60% in Yumin were born in Chabuchaer Maternal and Child Health Care Institute and Yumin County Hospital respectively. Maternal and Child Health Care Institutes in this study were woman's hospitals and also child health care centres. In Shihezi People's Hospital, mothers were recruited every second days due to due to staffing constraints. In other hospitals, all mothers were invited to the study and 97% (1219) agree to participate. Urumqi is the capital city of Xinjiang where the Uygur ethnic group is in the majority, while Shihezi is a predominantly Han ethnic area. Chabuchaer and Yumin counties have a larger concentration of Kazakh people and other minorities.

The majority of participants, including the minority groups, could read and speak Chinese (Mandarin). The questionnaire was originally prepared in Mandarin, and was also translated into the Uygur language, which can also be understood by Kazakh mothers. For those who could not read Chinese, trained nurses, who were fluent in the ethnic languages, were available to help them complete the questionnaires. For all minority mothers, follow up calls and visits were made in their own ethnic languages by nurses from their own ethnic group.

The questionnaire was based on those developed by Scott, Binns and Duong that have been extensively used in breastfeeding cohort studies in Australia, Vietnam and Kenya [17-22]. The questionnaires were designed to identify the feeding method and to collect information on factors associated with breastfeeding. After translation the questionnaires were tested in focus groups to ensure cultural appropriateness.

The project was approved by the Xinjiang local research authorities (Shihezi University, Urumqi Science Research Committee) and the Human Research Ethics Committee of Curtin University, Australia. Mothers who agreed to participate in the study signed the consent page in front of the questionnaire and were informed of their rights to withdraw from the follow up process at anytime without prejudice.

The sample size needed was calculated to be 860 to give a difference of eight percentage points in breastfeeding rates between the Han and Uygur groups at six months. (confidence 95%, power 80%), assuming the breastfeeding rate to be 75% in the Han Chinese. Additional numbers were included because of the other ethnic groups in the sample. All data analyses were carried out using the Statistical Package for Social Science (SPSS), release 12.0 (SPSS Inc., Chicago, IL, USA). Descriptive statistics and cross tabulations were generated for demographic variables, life tables were used for breastfeeding rates and Kaplan-Meier model was used to calculate mean of 'exclusive breastfeeding' and 'full breastfeeding' duration and assessed the differences in different ethnic groups. 'Significant' means p value less than 0.05.

The definitions of breastfeeding used in this paper are: [23-25]:

'Any breastfeeding': The child has received breastmilk (direct from the breast or expressed) with or without other drink, formula or other infant food.

'Exclusive breastfeeding': Breastfeeding while giving no other food or liquid, not even water, with the exception of drops or syrups consisting of vitamins, mineral supplements or medicine.

'Predominant breastfeeding': In addition to breastmilk the infant may receive small amounts of culturally valued supplement – water, water-based drinks, fruit juice, and ritualistic fluids.

'Full breastfeeding' includes 'exclusive breastfeeding' and 'predominant breastfeeding'.

## Results

The details of the sample and the distribution of the major demographic variables are shown in Table 1. The sample (1219) as recruited included 47% (578) Han, 30% (360) Uygur, 23% (281) other minorities.

Almost all of the mothers (1118) in the study were married, with eight separated and one widowed. The mean durations of hospital stay in days were 6.5 days (standard deviation 2.9) for Han Chinese, 6.1 days (SD 1.8) for Uygur and 6.4 days (SD 3.8) for the other ethnic groups. None of these results were significantly different from each other at the p < 0.05 level.

The breastfeeding rates were calculated by life table analysis and are detailed in Table 2. The breastfeeding rates on discharge were 89% in Han, 94% in Uygur and 97% in other ethnic groups and by six months these had declined to 77%, 55% and 88% respectively.

Inevitably in cohort study some mothers were lost to follow-up (Table 2). The percentage of mothers lost to follow-up at 0.5, 1.5, 2.5 3.5, 4.5 and 6 months were 6%, 7%, 9%, 12%, 13% and 21% respectively, that is 79% of mothers were followed to six months. This represents a response for 87.4% of the 'person-months' in the study.

**Figure 1:**
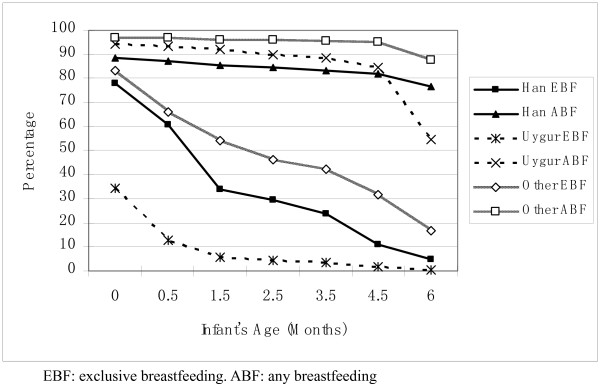
Breastfeeding Rates(%) in Han Ethnic, Uygur and 'Other Minority' Groups, Xinjiang, PR China, 2003–2004.

Figure 1 shows the 'any breastfeeding' and 'exclusive breastfeeding' rates by months for the different ethnic groups. Uygur babies were least likely to be 'exclusive breastfed' while the 'other minority' group had the highest 'any breastfeeding' and 'exclusive' breastfeeding rates.

Table 3 showed the duration of 'exclusive breastfeeding' and 'full breastfeeding' among ethnic groups. The overall 'exclusive breastfeeding' median was 0.5 month and 'full breastfeeding' median 2.5 months.

The censored rate at six months of 'exclusive breastfeeding' in Uygur, Han and other ethnics were 1.4%, 14.0% and 27.8% respectively, 'full breastfeeding' 9.7%, 18.9% and 34.2% respectively, and 'any breastfeeding' 72.5%, 81.0% and 91.8% respectively. Censored cases refer to those still breastfeeding or who had been lost to follow-up at six months.

Log-rank tests were run to determine if the duration of 'exclusive breastfeeding' and ' full breastfeeding' differ significantly between pairs of the ethnic groups, namely Uygur, Han and 'other minority' group. There were significant differences in the durations of 'exclusive breastfeeding' between the pairs among the ethnic groups. The 'other minority' group had the longest duration of 'exclusive breastfeeding', Uygur the lowest and Han in the middle (p < 0.01). The same trend was in 'full breastfeeding' rate (p < 0.01). The 'any breastfeeding' median duration was greater than six months in all ethnic groups. As follow-up in this study was terminated at six months, the maximum duration of breastfeeding could not be determined.

Uygur babies had the shortest 'exclusive and full breastfeeding' duration, the 'other minority' babies the longest and Han babies in the middle of the three groups. By four months of age, 66% of all infants had been commenced on solid foods. The percentage in Han, Uygur and 'other minority' groups were 71%, 85% and 34% respectively. At six months, the overall percentage was 78%, including 85% in the Han group, 91% in Uygur and 48% in the 'other minority' group. The differences between the groups were significant.

The factors likely to be associated with 'any breastfeeding' to six months were entered into Cox Regression model and are detailed in Table 4. Initially 21 factors were used in the model and two were independently associated with 'any breastfeeding' rate at six months.

## Discussion

This is the first reported longitudinal study of breastfeeding in the Xinjiang province. The minority mothers include Kazakh, Hui and Xibe were more likely to breastfeed their babies than Han mothers. Their 'any breastfeeding', 'full breastfeeding' and 'exclusive breastfeeding' rates were higher and duration of 'exclusive breastfeeding' and 'full breastfeeding' were longer than Han ethnic group. The results are a little different with a survey in Karamay, a medium sized city in North Xinjiang, in 1999 [26]. In that retrospective study, 'exclusive breastfeeding' rate at four months was 64% in Han and 69% in minorities (include Uygur, Kazakh and Mongolian) but the differences did not reach significant levels. The 'ever breastfed' rate in both Han and minority groups was 96%. However the time (mean month) of introduction of solid foods was significantly later in minority group (six months) than Han (five months)[26].

Uygur mothers were least likely to breastfeed their babies exclusively. This result was similar to the results from studies in Tibet, another minority area in West China, where the 'full breastfeeding' rate at the end of the first month was 56% (both urban and rural areas) and in 4–6 months declined to 3.0% in urban areas and 12.8% in rural areas [12]. In the present study, the 'full breastfeeding' rates in Uygur babies were 47% at the first month, declining to 1% at six months. The traditional beliefs about breastfeeding of the minority mother's may be responsible for the low rate of exclusive breastfeeding. Many Uygur mothers would state: 'breastmilk was not thick enough for baby's growth' as the reason for the early introduction of solids. Tibetan mothers were also used to feeding babies water early because they want to follow their tradition feeding method [12]. However the early introducing of water or complementary foods may lead to shortening of breastfeeding duration and exposes the infant to increased rates of morbidity and mortality [27].

In this study, 'exclusive breastfeeding' and 'full breastfeeding' rates were low and their durations were short in all ethnic groups. They were well below Chinese and international targets [8,28,29]. Besides traditional inappropriate perceptions, the advertising and ready availability of infant formula could be factors in the early introduction of additional foods to some infants. This is reflected in the lower rates of 'exclusive breastfeeding' by mothers living in urban areas who enjoy higher incomes [12]. Inappropriate infant feeding advice given by health professionals was another factor influencing 'exclusive breastfeeding'. Many doctors thought that infant jaundice was associated with dehydration and so they suggested feeding water to babies they considered at risk. In reality an early first breastfeed and frequent breastfeeds with no restrictions help to prevent or reduce jaundice [30].

More mothers in 'other ethnic group' lived in rural areas and have lower income. The formula was not so easy to get for them as the mothers living in urban areas with more income. So 'any breastfeeding' and 'exclusive breastfeeding' rates were higher than Han and Uygur groups. Some studies showed that maternal smoking was negatively associated with breastfeeding duration [31-34]. In Xinjiang, the paternal smoking rate was 64.8% but only a few mothers smoke (1.7%).

The main problem of breastfeeding in different ethnic groups in Xinjiang was the low prevalence of 'exclusive and full breastfeeding' rates. Mothers, especially Uygur mothers, need to be educated about the benefits of 'exclusive breastfeeding' for the first six months. However no specific factors, other than language, were identified to justify separate breastfeeding interventions specially targeted at Uygur or other minorities in Xinjiang. Further research, including qualitative studies are needed to provide a deeper understanding of the reasons for the early introduction of liquids and solid foods to infants who are being breastfed.

There are several limitations that need to be considered when interpreting the results of the study. As follow-up in this study was terminated at six months, the median duration of 'any breastfeeding' could not be determined. However the survival rates at six months still show the differences in ethnic groups. Further study is needed to identify factors associated with breastfeeding rate and duration in different ethnic groups. The proportion of mothers who could be contacted on follow up gradually declined over the duration of the project and at six months 79% of mothers provided information. The overall response in terms of person months in the study was 87.4% and these response rates need to considered when interpreting the study.

## Conclusion

The breastfeeding rates on discharge from hospital in Xinjiang were 88.5 %, 94.3 % and 97.1 % respectively, at six month 76.7 %, 54.7 % and 87.6 % respectively. However, 'exclusive breastfeeding' rates in Han, Uygur and 'other minority' groups at discharge were 78.0 %, 34.5 % and 83.1 % respectively, at six months 4.8 %, 0.4 % and 16.8 % respectively. Uygur babies were least likely to be 'exclusive breastfed'.

## Competing interests

The author(s) declare that they have no competing interests.

## Authors' contributions

FX: project design, data collection, data analysis and paper writing.

CWB: project design, data analysis and paper writing and revision.

GN: data collection and paper writing.

LS: data collection and paper writing.

YZ: data analysis.

AHL: data analysis.

## Pre-publication history

The pre-publication history for this paper can be accessed here:



## Figures and Tables

**Table 1:** Demographic Details of Han, Uygur and 'Other Minority' Groups in the Sample from Xinjiang, PR China, 2003–2004 (n = 1219)

Variable	Value	Han		Uygur		Others		Total	
		n	%	n	%	n	%	n	%
Place*	Urban Area	490	84.8	334	92.8	85	30.2	909	74.6
	Rural Area	88	15.2	26	7.2	196	69.8	310	25.4
	Missing	0		0		0		0	

Gestation Age* (weeks)	< 37	10	2.1	21	7.8	8	3.6	39	4.1
	37+	456	97.9	248	92.2	213	96.4	917	95.9
	Missing	112		91		60		263	

Birth Weight*	<2500 g	12	2.2	21	6.1	4	1.5	37	3.2
	2500–3999 g	496	88.9	285	82.4	238	90.2	1019	87.2
	4000 g+	50	9	40	11.6	22	8.3	112	9.6
	Missing	20		14		17		51	

Delivery Method*	Vaginal Delivery	243	45.2	173	50.4	186	72.1	602	52.9
	Caesarean	295	54.8	170	49.6	72	27.9	537	47.1
	Missing	40		17		23		80	

Birth Order*	1	456	87.7	265	77.5	203	77.8	924	82.3
	2+	64	12.3	77	22.5	58	22.2	199	17.7
	Missing	58		18		20		96	

Baby's Gender	Male	287	51.2	191	54.3	138	52.3	616	52.3
	Female	274	48.8	161	45.7	126	47.7	561	47.7
	Missing	17		8		17		42	

Maternal Job*	House Wife	191	35.6	169	49.1	140	53.2	500	43.7
	Sales	119	22.2	15	4.4	21	8	155	13.5
	Worker	76	14.2	37	10.8	6	2.3	119	10.4
	Farmer	54	10.1	8	2.3	33	12.5	95	8.3
	Office Worker	77	14.3	111	32.3	61	23.2	249	21.8
	Others	20	3.7	4	1.2	2	0.8	26	2.3
	Missing	41		16		18		75	

Maternal Age* (years)	<25	67	13	64	19.2	53	21	184	16.7
	25–29	260	50.4	157	47.1	127	50.4	544	49.4
	30–34	160	31	85	25.5	62	24.6	307	27.9
	35+	29	5.6	27	8.1	10	4	66	6
	Missing	62		27		29		118	

Maternal Education* (years)	< 9	198	37.8	133	38.8	145	53.9	476	41.9
	10–12	166	31.7	74	21.6	65	24.2	305	26.8
	13+	160	30.5	136	39.7	59	21.9	355	31.3
	Missing	54		17		12		83	

Family Annual Income* (RMB)	<10000	114	28.1	79	27.8	116	49.6	309	33.5
	10000–19999	171	42.2	113	39.8	72	30.8	356	38.6
	20000+	120	29.6	92	32.4	46	19.7	258	28
	Missing	173		76		47		296	

Family Members*	3	383	74.4	213	64	136	51.5	732	65.8
	4	67	13	93	27.9	49	18.6	209	18.8
	5	49	9.5	20	6	55	20.8	124	11.2
	6+	16	3.1	7	2.1	24	9.1	47	4.2
	Missing	63		27		17		107	

Paternal smoking	No	164	33.7	118	34.7	101	38.5	383	35.2
	Yes	322	66.3	222	65.3	161	61.5	705	64.8
	Missing	92		20		19		131	

Notes:

1. The 'other ethnic' group (n = 281) included 199 were Kazakh, 56 were Xibe and 26 Hui babies.

2. 100 Renami (RMB or CNY) is the equivalent of 12.5016 USD, 9.91190 EURO or 6.85308 GBP (Date of conversion 27 June 2006)

3. There were significant differences in all of the groups marked * (P < 0.01)

**Table 2:** Breastfeeding Rate (%) in Han Ethnic, Uygur and 'Other Minority' Groups, Xinjiang, PR China, 2003–2004

Ethnic	Month	n	Exclusive breastfeeding	Full breastfeeding	Any Breastfeeding
Han	0*	578	78.0	83.7	88.5
	0.5	534	61.0	71.7	87.2
	1.5	530	33.9	60.2	85.6
	2.5	523	29.4	55.1	84.5
	3.5	516	24.0	43.9	83.1
	4.5	502	10.9	14.8	82.0
	6	472	4.8	5.8	76.7

Uygur	0*	360	34.5	90.3	94.3
	0.5	341	12.6	47.2	93.4
	1.5	336	5.6	35.3	91.9
	2.5	317	4.5	22.1	90.0
	3.5	305	3.7	11.2	88.6
	4.5	302	1.9	3.4	84.8
	6	260	0.4	1.1	54.7

Others	0*	281	83.1	95.0	97.1
	0.5	272	65.9	79.9	96.7
	1.5	270	54.1	72.1	96.0
	2.5	265	46.2	61.5	96.0
	3.5	254	42.4	51.7	95.6
	4.5	251	31.9	37.9	95.2
	6	232	16.8	21.3	87.6

0 month refers to the time at discharge from hospital, which generally in one week postpartum.

The 'other ethnic' group (n = 281) included 199 were Kazakh, 56 were Xibe and 26 Hui babies.

**Table 3:** Median breastfeeding (interquartile range) duration in (months) Han, Uygur and other ethnic groups in Xinjiang, PR China, 2003–2004

	Exclusive Breastfeeding				Full Breastfeeding		
Ethnic group	N	Median	25%	75%	0.5	25%	75%

Uygur	360	0.1	0.5	0.1	0.5	2.5	0.5
Han	578	1.5	3.5	0.5	3.5	4.5	0.5
Others**	281	2.5	6.0	0.5	4.5	*	1.5

* The duration could not be calculated because censored percentage of 'full breastfeeding' in Other Group is 34% at six months

** The 'other ethnic groups (n = 281) included Kazakh 199, Xibe 56 and 26 Hui babies.

**Table 4:** Factors Associated With the Risk Discontinuing 'Any Breastfeeding' to Six Months, Xinjiang, PR China, 2003–2004

Factors	Values	Cox Regression			
		
		n	HR	95% CI	
Ethnics	Han	40	1		
	Uygur	164	2.93	1.14	7.56
	Others	63	0.94	0.31	2.88

Age of Infant When Mother Returned to Work	No Job	77	1		
	>= 6 Months	143	1.69	0.80	3.59
	< 6 Months	47	3.57	1.61	7.89

2 log likelihood (deviance) = 630.94, df = 4.

Non-significant variables were birth seasons, maternal age, job, years of education, delivery method, whether demand fed, time of making decision about infant feeding method, the time of baby's first suckling after birth, baby's first feed, how early breastmilk 'came in', whether maternal mother breastfed her babies, maternal mother's feeding preference, baby's gender, the gender mother prefer, baby's birthweight, mother's knowledge of formula, number of family members, whether pacifier used, whether mother able to breastfeed after returning to work.

All variables in the final model were variables for which, when excluded, the change in deviance compared with the corresponding chi-square test statistic on the relevant degrees of freedom was significant.

HR is Hazard Ratio and 95% CI is 95% confidence intervals.
